# Exploration of Natural Adsorbents for Applications in Pollution-Reducing Cosmetic Formulations

**DOI:** 10.3390/gels12030232

**Published:** 2026-03-12

**Authors:** Greta Kaspute, Alma Rucinskiene, Arunas Ramanavicius, Urte Prentice

**Affiliations:** State Research Institute Center for Physical Sciences and Technology, Sauletekio Av. 3, LT-10257 Vilnius, Lithuania; greta.kaspute@ftmc.lt (G.K.); alma.rucinskiene@ftmc.lt (A.R.); arunas.ramanavicius@chf.vu.lt (A.R.)

**Keywords:** gel-based natural adsorbents, gels, pollution-reducing cosmetics, skin, air pollution

## Abstract

Human skin and hair act as multifunctional barriers but are highly sensitive to environmental pollutants originating from air, water, and cosmetic products. Epidemiological studies report that exposure to particulate matter (PM_2.5_–PM_10_), nitrogen oxides (NO_x_), and volatile organic compounds increases the risk of skin and hair disorders. For instance, women in high-traffic areas (N = 211) show significantly more pigment spots and nasolabial wrinkles compared to those in rural areas (N = 189), indicating accelerated skin ageing. Children aged 9–11 exposed to PM_10_, benzene, and NO_x_ exhibit increased incidence of atopic dermatitis. Systemic exposure to dioxins causes chloracne, while co-exposure to polycyclic aromatic hydrocarbons (PAHs) and UVA radiation elevates skin cancer risk. Psoriasis flares are associated with mean pollutant concentrations over the 60 days preceding flare events in 957 patients, and hyperpigmentation prevalence increases in populations exposed to traffic-related PM and ROS-inducing pollutants. Hair loss is linked to oxidative stress from PM and PAHs absorbed on hair fibers, with in vitro studies showing keratinocyte apoptosis in scalp hair follicles. This review evaluates natural adsorbents such as zeolites, clays, activated carbon, and polyphenol-rich plant extracts for anti-pollution cosmetic formulations. Adsorption capacities range from 60 to 150 mg·g^−1^ depending on the pollutant, with removal efficiencies of 30–55% in model topical systems. Mechanisms include ion exchange, surface adsorption, hydrophobic interactions, and radical scavenging. Incorporating 2–5% *w*/*w* of these adsorbents in cosmetic formulations significantly reduces pollutant deposition on skin and hair. These findings support the development of evidence-based, sustainable anti-pollution cosmetic strategies that quantitatively mitigate environmental stressor effects.

## 1. Introduction

By 2030, one in every six people worldwide will be aged 60 or older. Additionally, the number of people aged 80 or older is anticipated to triple from 2020 to 2050, reaching 426 million [1]. Older adults often face skin issues due to aging, polypharmacy, and multimorbidity, which can affect their physical health, well-being, and quality of life [2]. In parallel, the global skincare market is projected to reach USD 230–250 billion by 2030, driven in part by rising consumer awareness of environmental stressors and the demand for protective cosmetic products [3]. This growing market provides an opportunity to develop targeted anti-pollution skincare and haircare solutions.

Skin is the outermost barrier of the body, which is sensitive to pollutants and is frequently exposed to the environment [4]. Skin accumulates toxic substances from jewellery, air, water pollutants, and makeup [5]. Chronic exposure to such pollutants accelerates intrinsic and extrinsic aging processes, leading to drier, less elastic skin with decreased sebum production, higher susceptibility to infections, inflammatory conditions, and increased risk of skin cancers [2].

Urbanization and industrialization have intensified these risks, as traffic fumes, cigarette smoke, and industrial emissions contribute significantly to oxidative stress, metabolic impairments, inflammation, and pigmentary disorders [4]. Hair is similarly affected, with pollutants adhering to hair fibers, inducing scalp irritation, dandruff, faster exfoliation, and potentially triggering psoriasis upon prolonged exposure [4].

The cosmetics industry has increasingly focused on addressing these environmental challenges, emphasizing formulations that restore the skin barrier, improve hydration, control pigmentation, reduce oxidative inflammation, and prevent collagen and elastin degradation [6]. Natural adsorbents—such as clays, zeolites, activated carbons, and plant extracts—offer promising strategies due to their low cost, high adsorption capacity, and abundance of bioactive metabolites [7,8]. On a molecular level, adsorption is when attractive forces associate a solute (adsorbate) with a solid surface (adsorbent). The solid material used for the adsorption consists of a porous medium with a high internal surface area [9]. For example, adsorption has been proven to be the best water treatment process. Clays and their minerals are abundant and cheap, removing toxic heavy metals from aqueous solutions [10]. As a result, anti-pollution cosmetics should contain these compounds to adsorb heavy metals, particulate matter (PM), gasses, and smoke from the skin. Various gel-based formulations are frequently used as water and other adsorbing material carriers. The development of gel-based natural adsorbents represents a promising and strategically important advancement in pollution-reducing cosmetic formulations [11]. By integrating natural adsorbents into cosmetic products, it is possible to develop evidence-based anti-pollution skincare solutions that mitigate environmental damage and slow the skin aging process.

The objective of this review is to systematically evaluate the potential of natural adsorbents as functional ingredients in anti-pollution cosmetic formulations for skin and hair protection. Specifically, this work (i) examines the impact of major environmental pollutants on skin and hair health, (ii) compares the adsorption performance and mechanisms of key natural materials such as zeolites, clays, activated carbon, and polyphenol-rich plant extracts, (iii) analyzes their incorporation into topical cosmetic systems and associated pollutant-removal efficiencies, and (iv) identifies current limitations, safety considerations, and future research directions for the development of effective and sustainable anti-pollution cosmetic products. This review uniquely integrates the dermatological impacts of environmental pollutants with the role of natural adsorbents and antioxidant-based ingredients, highlighting their combined application in gel-based anti-pollution cosmetic formulations. This review focuses on adsorption-driven strategies in anti-pollution cosmetics, highlighting how natural adsorbents and gel-forming materials can actively sequester pollutants at the skin and hair interface, providing a mechanistically unified framework distinct from antioxidant- or essential oil-based approaches. Unlike previous reviews that focus solely on antioxidants or pollutant types, this manuscript provides a comprehensive, translational perspective linking skin biology, material science, and cosmetic innovation.

## 2. Air Pollution as a Driver for Anti-Pollution Cosmetic Development

Air pollution has emerged as a major extrinsic factor accelerating skin ageing and barrier dysfunction, creating new challenges for preventive dermatology and cosmetic science. From a formulation perspective, the increasing burden of particulate matter (PM), polycyclic aromatic hydrocarbons (PAHs), heavy metals, and volatile organic compounds (VOCs) highlights the need for cosmetic systems that not only moisturize the skin but also actively limit pollutant deposition and penetration [12,13,14]. Consequently, understanding pollution–skin interactions is essential for the rational design of functional excipients, particularly natural adsorbents, in next-generation anti-pollution cosmetic products.

Ambient air pollution is a complex mixture of solid particles and gaseous components originating from traffic emissions, industrial activity, domestic heating, and natural sources [15]. Among these, fine particulate matter (PM_2.5_ and PM_10_) is of particular concern due to its small size, large surface area, and ability to carry adsorbed toxic compounds. These particles readily deposit on exposed skin and hair surfaces, where they may penetrate through hair follicles, sweat ducts, or compromised stratum corneum regions [4,15].

**Figure 1:**
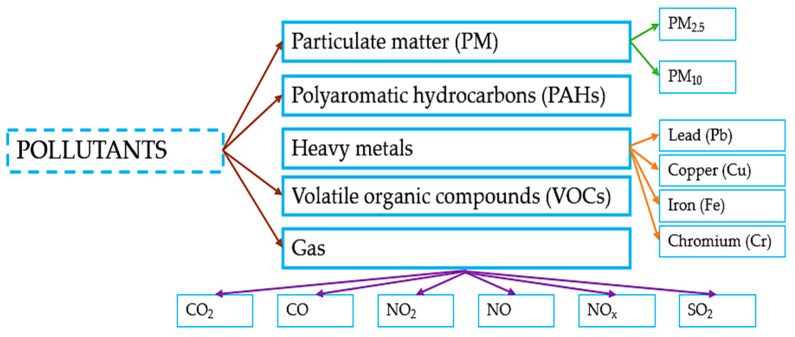
Types of air and water pollutants [4,15]. The scheme represents the central air and water pollutants which affect the human body.

Mechanistically, pollutant exposure induces skin damage through several interconnected pathways. PM and PAHs can activate the aryl hydrocarbon receptor (AhR), triggering oxidative stress and inflammatory signaling [15]. Heavy metals such as lead and cadmium catalyze reactive oxygen species (ROS) formation [10], while ozone and VOCs promote lipid peroxidation and depletion of endogenous antioxidants. Collectively, these processes contribute to premature skin ageing, hyperpigmentation, impaired barrier function, and exacerbation of inflammatory dermatoses [16] (Figure 1).

Importantly, many pollutants initially accumulate at the skin and hair surface before deeper penetration occurs [8]. This interfacial stage represents a critical intervention window for topical products. Cosmetic formulations that incorporate adsorptive or film-forming excipients may reduce the residence time and bioavailability of pollutants on the skin. Therefore, pollution-associated dermatological outcomes are directly relevant to cosmetic formulation strategies aimed at surface protection and pollutant sequestration [17,18].

Epidemiological and experimental studies have associated chronic pollution exposure with increased wrinkle formation, pigment irregularities, dryness, scalp irritation, and hair fiber damage [6]. In urban environments, repeated deposition of PM on hair shafts can lead to cuticle roughness, decreased shine, and increased fragility [19,20]. These observations further support the need for multifunctional cosmetic ingredients capable of both protecting biological tissues and improving sensory performance.

Table 1 summarizes major pollution-related skin and hair conditions that provide the biological rationale for incorporating natural adsorbents into anti-pollution cosmetic systems. By physically binding pollutants at the formulation–skin interface, such materials may complement traditional antioxidant and barrier-repair approaches.

Collectively, current evidence indicates that modern cosmetic formulations must move beyond passive hydration and photoprotection toward active pollutant-mitigation strategies. This shift has stimulated growing interest in multifunctional excipients—particularly natural adsorbents—that can reduce pollutant burden at the skin surface while maintaining safety, sustainability, and consumer acceptability.

## 3. The Main Goals of Anti-Pollution Cosmetics

Many skincare strategies focus on removing pollutants from the skin and enhancing antioxidant defences, while new formulations aim to strengthen the skin barrier and support microbiota health. The use of plant extracts, fermented plant materials, and probiotic cultures in skincare products has shown promising results in combating oxidative damage and inflammation caused by air pollution [35]. Active ingredients used in anti-pollution skincare products aim to prevent and alleviate the effects of environmental stressors like PM [36]. The anti-pollution cosmetics trend originated in Asia and subsequently spread to Western markets and all over the world. The demand for natural actives is growing in all segments of the global cosmetic market, i.e., anti-pollution cosmetics [8,37]. Several cosmetic strategies can be adopted to protect human skin against environmental pollution, e.g., an effective cosmetic anti-pollution routine to remove chemicals deposited on the skin or the isolation of the epidermis through the formation of a cohesive and non-occlusive film on its surface, preventing the direct contact with airborne pollutants [8]. Anti-pollution ingredients are often categorized based on their mechanisms of action. Some ingredients help prevent the deposition of pollutants on the skin, while others focus on removing them. Surfactants and film-forming polymers play a key role in these products, which can come in various forms such as cleansers, masks, moisturizers, and wipes [36]. For cleaning purposes, materials like activated charcoal, kaolin, magnesium aluminium silicate, coffee beans, and rice bran are often used. These ingredients help adsorb pollutants from the skin [36].

Also, it is important to use sun protection, which is essential for the prevention of photo-reactive compounds that cause photo-pollution skin damage, i.e., using products with broad-spectrum (UVA + UVB) sun protection, sunscreens with the addition of antioxidants (vitamin E, ferulic acid, etc.), and sunscreens and after-sun lotions containing DNA repair enzymes [6]. Daily application of skincare products can be an effective shield against polluting agents. Anti-pollution hair cosmetics work through film forming and anti-adhesive effects. The film blocks pollutant particle adsorption and can be removed easily after rinsing [4]. The physical barrier for the pollutant can be obtained using film-forming ingredients, both synthetic (silicones, acrylic acid copolymers) and naturally derived (peptides and polysaccharides extracted from plants or obtained by fermentation processes). There is a need for the inclusion in anti-pollution formulations of antioxidants, in order to protect against ROS effects, or ingredients able to up-regulate the antioxidant defences of the epidermis cells [8].

### 3.1. Natural-Derived Molecules to Regulate Aging Mechanism

Plant extracts and other natural compounds with strong antioxidant properties can help protect and treat oxidative skin damage caused by air pollution [38]. Notably, a topical antioxidant blend, which includes 19 different water-soluble, lipid-soluble, and enzymatic antioxidants, is designed to shield the skin from UV radiation and reduce visible signs of photodamage. This mixture has been shown to reduce oxidative stress caused by blue light, cigarette smoke, and ozone exposure in skin models [6]. A serum containing *Deschampsia antartica* extract, ferulic acid, and vitamin C was evaluated in a 28-day study to assess its protective effects against air pollution-induced skin damage [39]. The study involved 20 women living in a high-pollution urban area and showed significant improvements in skin hydration, reduction in dark spots, and a reduction in oxidative stress markers. The serum also demonstrated a positive cosmetic acceptability, with over 90% of participants expressing satisfaction. These findings suggest that the serum effectively enhances skin barrier function and counters the detrimental effects of pollution [39]. Another study investigated the protective effects of formulations containing antioxidants or a chelating agent against skin damage from pollution, specifically cigarette smoke. Antioxidant epigallocatechin gallate provided the best protection against radicals and oxidative stress, with the chelating agent ethylenediamine-N,N′-disuccinic acid also showing notable effects [40].

Tapinarof is a novel, naturally derived small-molecule agent undergoing clinical trials for treating atopic dermatitis and psoriasis, working by activating AhR in the skin to reduce inflammation [6]. It works by binding to and activating AhR, helping to reduce skin inflammation, restore the skin barrier, decrease oxidative stress, and regulate gene expression in immune cells [41]. Topical application of tapinarof has been shown to decrease cytokine levels, epidermal thickening, and erythema in mice with psoriasiform lesions, though it has no effect in AhR-deficient mice. Additionally, tapinarof has antioxidative properties, activates Nrf2, and downregulates inflammatory cytokines like IL-17 and P-STAT6, making it a promising treatment for AD and potentially air-pollution-related skin flare-ups [6]. Interleukin (IL)-37 plays a role in suppressing both systemic and local inflammation and is expressed in the epidermis, with levels decreased in inflammatory skin diseases like AD and psoriasis. A topical agent that can increase IL-37 expression could potentially be effective for treating these conditions, though the regulatory mechanisms of IL-37 remain unclear. It was found that treatment with tapinarof and Galactomyces ferment filtrate, both AhR modulators, increased IL-37 levels in human keratinocytes, and this effect was reversed when AhR was knocked down. Additionally, IL-37 was shown to inhibit the expression of IL-33, a cytokine implicated in AD and psoriasis, suggesting that the AhR/IL-37 pathway could be targeted to prevent IL-33-related skin disorders [42]. Clinical trials of tapinarof cream have confirmed that AhR agonism is an effective therapeutic strategy for treating inflammatory skin conditions. Tapinarof 1% cream is a first-of-its-kind, nonsteroidal, topical AhR agonist with a pharmacokinetic profile that ensures localized treatment at affected areas, minimizing concerns about systemic safety, drug interactions, or off-target effects [43].

Essential oils (EOs) and their components have long been used in traditional medicine and aromatherapy to manage various health conditions, with their use continuing to grow in recent times [44,45]. Numerous studies have explored the neuroprotective and anti-aging potential of EOs, investigating their mechanisms of action. EOs from plants such as *Nigella sativa*, *Acorus gramineus*, *Lavandula angustifolia*, *Eucalyptus globulus*, *Mentha piperita*, *Rosmarinus officinalis*, *Jasminum sambac*, and *Piper nigrum* have shown neuroprotective effects, particularly in relation to neurodegenerative disorders like Alzheimer’s disease and dementia [46]. A randomized controlled trial identified the impact of aromatherapy on dry skin in elderly individuals living in a nursing home. The results showed that the aromatherapy group experienced significant improvements in skin moisture levels across multiple areas, with the most noticeable improvements occurring after two weeks. In contrast, the olive oil using group also showed some skin moisture improvement, though to a lesser extent, while the control group showed no change in skin moisture, maintaining their dry skin condition [47]. Moisturizers help enhance skin hydration by promoting corneo-desmosome degradation, softening the skin, reducing friction, and sealing cracks, making them essential for treating xerosis and pruritus, particularly in the elderly. Those with antiseptic, antibacterial, and antioxidant properties are recommended for senile xerosis. In tropical regions, EOs have long been used as traditional moisturizers, effectively improving the epidermal barrier function and preventing transepidermal water loss [48]. The research aimed to evaluate the anti-aging effects and skin irritation potential of four EOs from Indonesia: patchouli, nutmeg, clove, and citronella. The anti-aging activity was assessed using both in vitro yeast viability tests for endogenous aging and in vivo rat skin models exposed to UVB for exogenous aging. The results showed that all four oils improved yeast viability at certain concentrations and reduced wrinkle formation in rats after UVB exposure. In terms of skin irritation, patchouli and nutmeg oils showed no irritation, citronella caused slight irritation, while clove oil was found to be very irritating [49].

Polysaccharides from plants have long been studied for their pharmaceutical benefits, and recent research is increasingly focused on their potential to improve human skin health through external applications [1]. New eco-friendly and sustainable polysaccharides are being developed, with *Dendrobium* spp., known for its bioactivities, gaining attention for its potential in skincare [50]. While research has predominantly focused on the stem polysaccharides of *Dendrobium* orchids, the flower polysaccharides have been largely neglected, despite a significant portion of the flowers being discarded. Recent eco-friendly methods have been developed to extract polysaccharides from low-quality *Dendrobium* flowers, showing potential in cosmetics for treating skin dryness without toxicity [51]. Similarly, polysaccharides extracted from red-ginseng by-products have been shown to prevent UV-induced skin aging and suppress atopic dermatitis, offering an opportunity to utilize 8000 tons of ginseng by-product annually in cosmetic formulations [52]. The study examined the effects of spray-dried algae-rosemary particles, specifically a gel made from Spirulina and rosemary extract, against pollution-induced skin damage. The complexation of rosemary extract with Chlorella and Spirulina protein powders was tested, with Spirulina showing higher levels of beneficial phenolic compounds. The ex vivo results demonstrated that the Spirulina–rosemary gel effectively reduced oxidative and inflammatory skin responses caused by diesel engine exhaust exposure. The gel’s application helped decrease harmful markers such as 4HNE-PA and MMP-9, as well as prevent filaggrin loss, suggesting its potential in preventing pollution-induced skin aging [53].

Although many natural polysaccharides show skin benefits, most research has not translated into successful commercial products, partly due to the complexity of extracting and purifying these substances. Additionally, biosafety concerns, such as cytotoxicity, have limited clinical studies on the skin protection abilities of natural ingredients [50]. Current studies often focus on discovering new ingredients without exploring the mechanisms behind their effects, and the structure–activity relationships of these polysaccharides remain underexplored. Comprehensive research into the purification, characterization, and toxicity of natural polysaccharides, as well as their mechanisms of action, is needed to better understand their skin benefits. Furthermore, the establishment of regulations for the sustainable use of novel natural resources and the development of standardized extraction processes are critical for the large-scale commercialization of these polysaccharides in the cosmetic industry [50].

Phenolic compounds, including flavonoids and non-flavonoid polyphenols, play a key role in protecting the skin from oxidative stress and inflammation induced by pollutants like PM [54,55]. Polyphenols such as quercetin and resveratrol exhibit strong antioxidant properties, reduce oxidative stress markers, and help protect skin through various mechanisms, including modulating inflammatory pathways and skin aging proteins [56,57,58]. Tea polyphenols and algal polyphenols further contribute to skin protection by scavenging free radicals, regulating oxidative stress markers, and inhibiting inflammatory cytokines [59,60,61]. Phytosterols, like fucosterol, also help reduce ROS production and inflammatory responses, offering protective effects against PM-induced skin damage [62,63,64]. Ginsenosides, particularly ginsenoside Rb1 from ginseng, show promise in mitigating oxidative stress and apoptosis in skin cells exposed to pollutants [65,66]. Niacinamide, a form of vitamin B3, aids in protecting skin cells from oxidative damage, while the ethanol extract of *Cornus officinalis* fruit offers antioxidant and anti-inflammatory effects that safeguard against PM-induced injury. Additionally, fish oil, rich in omega-3 fatty acids, has been shown to reduce oxidative damage and inflammation, offering potential protection against skin aging caused by environmental pollutants [67].

The bioactivity of natural-derived molecules in anti-aging and anti-pollution skincare is strongly linked to their chemical structure and functional groups. This information is presented in Table 2.

### 3.2. Natural Adsorbents as Ingredients for Natural Cosmetics

Natural adsorbents, or biosorbents, are derived from materials such as plant biomass, volcanic rocks, soils, agricultural and industrial wastes, animal shells, microalgae, and fungal biomass. These materials possess large specific surface areas and diverse functional groups (e.g., carboxyl, hydroxyl, amino), enabling physical and chemical retention of pollutants, including heavy metals, particulate matter (PM), and volatile organic compounds (VOCs) [7]. Nanoporous inorganic materials, particularly zeolites, are natural or synthetic crystalline aluminosilicates with open three-dimensional frameworks that allow effective ion exchange and adsorption of pollutants [69]. Clinoptilolite, a widely studied zeolite, has demonstrated high adsorption capacities for Pb^2+^ (120–150 mg·g^−1^) and Cd^2+^ (95–130 mg·g^−1^), with efficacy depending on pH, contaminant type, and zeolite concentration (1–3% *w*/*w*) in cosmetic formulations [70]. Zeolites are mined from geological deposits in countries including the USA, China, and Eastern Europe, are cost-effective (<$1/kg), environmentally sustainable when extracted responsibly, and have been used since the 18th century [71].

Clays such as kaolinite and montmorillonite are naturally occurring layered silicates that effectively adsorb PM due to their high surface area and intercalation capacity [72]. They are globally abundant, inexpensive ($0.005–0.46/kg), and have a long history of use in water purification and cosmetic products [73]. Agricultural waste materials, including sawdust, *Moringa oleifera* bark, *Acacia nilotica* stems, and coconut or *Cinnamomum camphora* powders, offer additional functional groups capable of binding heavy metals through ion exchange or complexation mechanisms, presenting low-cost and sustainable alternatives for topical application [74].

Activated carbons derived from plant biomass, such as coconut shells and wood, adsorb VOCs via hydrophobic and π–π interactions, with capacities of 90–125 mg·g^−1^. Production involves carbonization and activation processes, making them moderately costly and energy-intensive, yet highly effective [75]. Polyphenol-rich plant extracts, obtained from leaves, seeds, or peels through solvent or enzymatic extraction, do not directly adsorb pollutants but mitigate pollutant-induced oxidative stress. Their radical scavenging capacities range from 95 to 140 mg·g^−1^, and they are biodegradable with low environmental impact [76].

When incorporated into topical formulations at 2–5% *w*/*w*, these adsorbents demonstrate measurable reductions in pollutant deposition: zeolite- or clay-based creams reduce PM_2.5_ or heavy metal accumulation by 35–50%, activated carbon formulations decrease VOC exposure by 45–55%, and polyphenol-rich extracts reduce oxidative species by 30–45% in ex vivo skin models [5,77]. Macromolecules in cosmetic formulations, such as cationic surfactants and polyelectrolytes, further enhance adsorption onto keratin substrates of hair and skin, improving pollutant capture and hair manageability [78].

**Figure 2:**
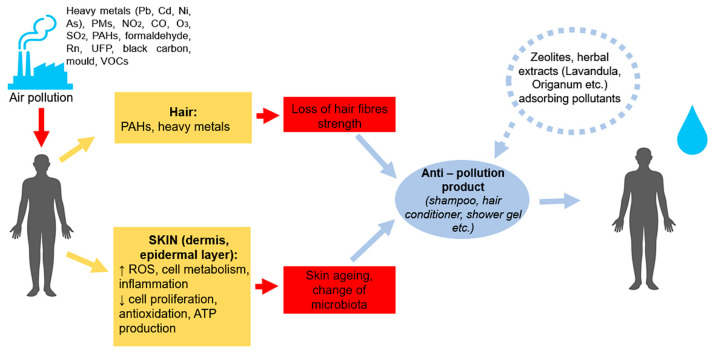
Opportunity to apply natural adsorbents for anti-pollution product development. This figure represents a schematic view of possible cosmetic product roles containing natural adsorbent’s role in pollution absorption. The product should be activated by water.

These mechanisms and material properties provide opportunities for innovation in anti-pollution cosmetic product design. Figure 2 illustrates how natural adsorbents can be incorporated into diverse product formats—such as shampoos, shower gels, conditioners, face masks, or creams—activated by water to capture pollutants on skin and hair. The figure highlights the potential multifunctionality of biosorbents, combining pollutant adsorption, barrier protection, and compatibility with daily-use formulations.

Table 3 summarizes adsorption capacities, pollutant removal efficiencies, origins, extraction methods, costs, environmental impact, and first known use of representative natural adsorbents. While biosorbents offer low operation costs, broad pollutant-binding capacities, and compatibility with multiple cosmetic formats, they also have limitations, including shorter lifetimes, early saturation, variability in raw material quality, and a lack of standardized testing protocols.

While natural adsorbents provide broad pollutant-binding capabilities, their effectiveness can be significantly enhanced when incorporated into gel matrices, which improve stability, skin adherence, and controlled sequestration, forming the basis for next-generation multifunctional anti-pollution formulations.

### 3.3. Natural Gel-Forming Materials for Cosmetics

Natural gel-forming materials, including polysaccharide-based hydrogels (e.g., alginate, chitosan, cellulose derivatives), protein-based gels, and plant-derived biopolymer networks, demonstrate strong potential for entrapping and immobilizing pollutants such as heavy metals, excess sebum, particulate matter, and residual organic contaminants [87]. Their three-dimensional crosslinked structures provide high surface area, tunable porosity, and controlled diffusion properties, enabling efficient adsorption while maintaining desirable rheological and sensory characteristics essential for topical cosmetic applications [88,89].

Gel networks can be formed through ionic crosslinking (e.g., alginate with Ca^2+^, chitosan with tripolyphosphate) or covalent crosslinking (e.g., enzymatic or chemical crosslinkers) [90]. Ionic gels typically exhibit reversible bonding and stimuli-responsive behavior, while covalent gels offer greater mechanical stability and long-term durability [91]. Porosity engineering—controlled through polymer concentration, crosslinking density, or incorporation of porogens—directly affects pollutant uptake, diffusion kinetics, and swelling behavior [92,93]. Swelling-controlled adsorption allows gels to sequester pollutants efficiently while gradually releasing moisture or active ingredients, supporting multifunctional cosmetic performance [94]. Biopolymers of interest include alginate, chitosan, cellulose derivatives, and protein-based polymers due to their capacity to interact with and sequester environmental pollutants. Alginate, a naturally derived polysaccharide obtained from brown algae, forms ionic gels in the presence of divalent cations and has demonstrated strong potential for heavy-metal chelation and particulate matter capture [95]. Chitosan, a positively charged polysaccharide derived from chitin, is particularly effective at adsorbing negatively charged pollutants and can enhance adherence to biological substrates such as hair and skin [96]. Cellulose derivatives, including hydroxypropyl methylcellulose and carboxymethylcellulose, contribute functional properties such as viscosity enhancement, barrier formation, and the physical entrapment of pollutants within polymer matrices [97]. In addition, gelatin and plant-derived proteins provide high biocompatibility and film-forming capability, while their controlled swelling behavior facilitates pollutant sequestration within hydrated polymer networks [98].

Importantly, gel-based systems offer several formulation advantages over powdered or particulate adsorbents. They reduce particle inhalation risks, enhance dispersion stability, improve skin adherence, and allow controlled release or sequestration mechanisms within the cosmetic matrix [99]. Furthermore, biodegradable gel networks align with circular economy principles, contributing to reduced post-use environmental burden compared to synthetic polymers or microplastic-based thickeners [100].

Recent advances in green crosslinking strategies, nanostructured biopolymer reinforcement, and hybrid gel composites have further enhanced adsorption capacity, mechanical stability, and compatibility with active cosmetic ingredients [101]. These innovations enable multifunctional performance combining adsorption, moisturization, antioxidant delivery, and barrier support within a single system [101,102].

Nevertheless, several challenges must be addressed to ensure successful translation into commercial products. These include long-term stability under varying storage conditions, preservation compatibility, large-scale reproducibility of natural polymers, regulatory compliance, and comprehensive ecotoxicological evaluation [103]. Additionally, systematic life cycle assessments are necessary to confirm the net environmental benefit of gel-based adsorbent systems in real-world cosmetic use and wastewater discharge scenarios [104].

Future research directions should prioritize: (i) optimization of gel network architecture for enhanced adsorption kinetics; (ii) development of stimuli-responsive or regenerable gel systems; (iii) evaluation of pollutant sequestration in simulated rinse-off and leave-on scenarios; (iv) integration of agricultural waste-derived biopolymers into gel matrices; (v) standardization of sustainability metrics for cosmetic-grade biogels.

## 4. Discussion

By combining insights from dermatology, natural adsorbent chemistry, and antioxidant mechanisms, this review establishes a unified framework for understanding how nature-derived ingredients can be strategically incorporated into anti-pollution cosmetics, emphasizing both efficacy and sustainability. This integrated approach positions the current work as a novel reference for researchers and formulators aiming to develop next-generation protective skincare

*Translational role of biosorbents in anti-pollution cosmetics*

Natural adsorbents and biosorbents have emerged as promising functional ingredients because of their capacity to bind particulate matter, heavy metals, and organic pollutants at the skin surface. Materials such as zeolites, clays, activated carbon, and polyphenol-rich plant extracts demonstrate measurable pollutant-reduction potential when incorporated into topical formulations [5,73,75,79,80,81,82,83,86]. Their advantages include broad adsorption spectra, relative abundance, and compatibility with multiple cosmetic formats.

From a formulation perspective, biosorbents can provide dual functionality: (i) physical adsorption of environmental contaminants and (ii) indirect biological protection through antioxidant and anti-inflammatory activity. Gel-based matrices are particularly attractive delivery systems because they enable uniform dispersion of adsorbent particles, controlled release of bioactives, favorable sensory properties, and enhanced skin residence time [88,89,90,91]. Compared with traditional emulsions, structured gels may improve pollutant capture efficiency while maintaining consumer acceptability.

However, important limitations remain. Mineral adsorbents may introduce impurities (e.g., trace heavy metals), exhibit batch variability, or negatively affect product aesthetics if not properly processed. Plant-derived biosorbents, while biologically attractive, often suffer from extraction complexity, stability issues, and insufficient mechanistic characterization. Moreover, most available studies rely on in vitro or short-term ex vivo models, limiting direct clinical translation.

*Clinical and public health relevance*

The increasing prevalence of pollution-related skin disorders has important implications for healthcare systems. Older adults, particularly those in institutional care, frequently exhibit xerosis, dermatitis, and barrier impairment that may be exacerbated by environmental stressors [24,41,53,91]. Preventive dermocosmetic strategies incorporating biosorbents could therefore contribute to reducing dermatological morbidity and associated healthcare costs.

From a health-economic perspective, the integration of low-cost natural adsorbents into widely accessible cosmetic products represents a potentially cost-effective preventive approach. By reducing pollutant-induced skin damage, such products may help decrease the long-term burden associated with chronic inflammatory dermatoses and premature skin aging. Nevertheless, robust cost-effectiveness analyses and real-world outcome studies remain scarce and should be prioritized in future research.

*Advantages and limitations of current applications*

Natural biosorbents offer several key advantages that make them promising candidates for anti-pollution cosmetic formulations. They exhibit broad pollutant-binding capacity, effectively targeting particulate matter, volatile organic compounds, and heavy metals. These materials are compatible with multiple cosmetic formats, including cleansers, masks, moisturizers, and mists, and can provide synergistic effects when combined with antioxidants or barrier-repair agents. When properly purified, biosorbents generally have a favorable safety profile and align well with consumer demand for natural and sustainable ingredients.

However, there are notable limitations that must be addressed. Long-term clinical evidence demonstrating sustained efficacy is limited, and variability in raw material quality and purity can affect performance. Some formulations may present sensory challenges, such as grittiness or whitening, and the structure–activity relationships of many natural adsorbents remain poorly understood. Additionally, standardized testing protocols are lacking, and regulatory pathways for novel bioactive claims are still uncertain. Balancing these strengths and weaknesses underscores the need for rigorous translational research to support the safe and effective integration of biosorbents into mainstream anti-pollution skincare products.

*Regulatory considerations and safety framework*

Successful commercialization of biosorbent-based anti-pollution cosmetics requires careful alignment with international regulatory requirements governing cosmetic safety and product claims. Regulatory authorities, including the U.S. Food and Drug Administration (FDA) and the European Commission, require that cosmetic products be demonstrably safe for their intended use and supported by appropriate scientific substantiation. As anti-pollution cosmetics often incorporate functional materials such as mineral adsorbents and plant-derived biosorbents, manufacturers must ensure that both ingredient safety and product claims comply with applicable regulatory frameworks.

In the United States, cosmetics are regulated under the Federal Food, Drug, and Cosmetic Act. Under this framework, cosmetic products do not generally require premarket approval, with the exception of certain color additives. However, manufacturers and distributors bear full legal responsibility for ensuring product safety, proper manufacturing practices, and truthful labeling. Importantly, regulatory classification may change if cosmetic products are marketed with claims suggesting prevention or treatment of dermatological conditions. In such cases, the product may be regulated as a drug, which introduces substantially stricter requirements for safety evaluation, efficacy demonstration, and regulatory approval.

Within the European Union, cosmetic products are governed by Regulation (EC) No 1223/2009 [105] on cosmetic products, which establishes one of the most comprehensive regulatory frameworks for cosmetic safety. This regulation requires the preparation of a Cosmetic Product Safety Report (CPSR), adherence to Good Manufacturing Practice (GMP), and detailed toxicological assessment of all ingredients used in the formulation. In addition, manufacturers must evaluate potential impurities, contaminants, and exposure levels, while ensuring that all product claims are supported by adequate scientific evidence. The regulation also mandates the maintenance of a Product Information File (PIF), which documents formulation data, safety assessments, and supporting evidence for regulatory compliance.

For mineral biosorbents such as clays and zeolites, regulatory evaluation often focuses on parameters including heavy-metal impurity levels, particle size distribution, and dermal safety. These factors are particularly important because mineral materials may naturally contain trace contaminants that must be controlled to ensure consumer safety. In contrast, plant-derived biosorbents present additional considerations related to batch-to-batch consistency, allergenicity, solvent residues from extraction processes, and microbiological quality. Ensuring consistent composition and purity is therefore essential for maintaining regulatory compliance and product safety.

Recent regulatory and industry trends further emphasize the importance of evidence-based substantiation of cosmetic claims, particularly for products marketed with “anti-pollution” benefits. Regulatory authorities increasingly expect robust experimental or clinical evidence demonstrating that formulations can effectively reduce pollutant deposition, mitigate oxidative stress, or support skin barrier function. At the same time, broader sustainability considerations—including environmental impact, responsible sourcing of natural ingredients, and compatibility with the skin microbiome—are gaining increasing importance within both regulatory guidance and consumer expectations. Transparency regarding ingredient origin and manufacturing processes is also becoming a key component of regulatory and market acceptance.

Consequently, integrating regulatory strategy into the early stages of product development is critical for facilitating the successful translation of biosorbent-based anti-pollution formulations from research concepts to commercially viable cosmetic products.

*Key research gaps and future priorities*

Despite significant advances in the development of biosorbent-based anti-pollution cosmetic formulations, several critical knowledge gaps persist that must be addressed to enable their clinical and commercial translation. One major challenge is the lack of standardized experimental models for evaluating pollutant exposure and adsorption efficiency. Without harmonized methodologies, comparisons across studies remain difficult, limiting mechanistic understanding and the generation of robust performance data. In parallel, well-designed clinical trials are needed to establish the long-term protective efficacy of these formulations under real-world conditions, particularly in urban environments where complex mixtures of particulate matter, volatile organic compounds, and heavy metals prevail.

Mechanistic clarification of the structure–activity relationships governing natural adsorbents is another priority. Understanding how polymer chemistry, surface charge, porosity, and hydrophilic/hydrophobic balance influence pollutant binding and skin interaction will inform rational formulation design. Similarly, optimization of gel-based delivery systems is required to enhance stability, sensory attributes, and functional performance, ensuring that biosorbents maintain activity throughout the product’s shelf life and during consumer use. Real-world exposure studies are also essential to evaluate efficacy against heterogeneous pollutant mixtures that are difficult to replicate in laboratory settings.

Beyond formulation and efficacy, comprehensive assessments of sustainability, environmental impact, and life-cycle considerations are increasingly important. Evaluating the ecological footprint of biosorbent production, processing, and disposal will be critical for aligning product development with sustainability goals and consumer expectations. Robust health-economic analyses are similarly needed to quantify the preventive value of anti-pollution cosmetics, supporting both commercial viability and public health relevance. Finally, clarifying regulatory pathways for novel biosorbent technologies—including safety evaluation, claim substantiation, and compliance with international frameworks—remains essential for successful market translation.

Addressing these interconnected research priorities will be vital for transitioning biosorbent-based cosmetics from promising experimental concepts to clinically validated, commercially feasible solutions that provide demonstrable protective benefits against environmental pollutants.

## 5. Conclusions

Natural biosorbents represent a promising class of functional ingredients for anti-pollution cosmetic and dermocosmetic formulations. Materials such as zeolites, clays, activated carbon, and polyphenol-rich plant extracts have demonstrated measurable capacity to adsorb environmental pollutants, including particulate matter, heavy metals, and organic contaminants, thereby reducing their potential interaction with skin and hair. When incorporated into structured delivery systems, particularly gel-based matrices, these materials may provide additional advantages such as improved formulation stability, enhanced pollutant sequestration, and the formation of protective barriers on biological surfaces.

Gel-based natural adsorbent systems therefore offer a multifunctional platform that integrates structural support, pollutant capture, and potential biological protection while maintaining compatibility with sustainability-driven cosmetic design. Their biodegradable nature and adaptability to diverse cosmetic formats further support their relevance in the development of next-generation anti-pollution products.

Despite encouraging in vitro and ex vivo findings, several challenges remain before these materials can be fully translated into widely adopted cosmetic technologies. Standardized evaluation methods, robust clinical validation, and clearer regulatory frameworks are necessary to confirm their real-world efficacy and safety. Future progress will require interdisciplinary collaboration among cosmetic scientists, dermatologists, toxicologists, materials scientists, and regulatory specialists to better understand adsorption mechanisms, optimize formulation strategies, and establish consistent performance benchmarks.

Continued research in these areas will be critical for enabling biosorbent-based dermocosmetics to evolve from experimental concepts into effective, scalable, and sustainable preventive strategies for mitigating pollution-related skin and hair damage.

## Figures and Tables

**Table 1:** Pollutant-caused skin or hair health problems.

Condition	Major Pollutants Involved	Key Biological/Clinical Mechanism	Implications for Anti-Pollution Cosmetics	Ref.
Dull, dry, lifeless hair and scalp	Dioxins, airborne particulate matter	Dioxins adhere to hair fibers via adsorption, indicating environmental exposure; pollutant deposition worsens hair appearance	Film-forming and anti-adhesive formulations can reduce particle attachment and facilitate removal during rinsing	[4,21]
Skin ageing (wrinkles, pigment spots)	Traffic-related particles, cigarette smoke	Pollution exposure associated with increased pigment spots and nasolabial wrinkles; smoking promotes keratinocyte dysplasia and elastosis	Need for formulations that limit pollutant deposition and oxidative stress	[6,22,23]
Atopic dermatitis (eczema)	PM_10_, benzene, NOx, CO, wildfire smoke	Air pollutants linked with increased AD incidence and itch; symptoms influenced by environmental factors	Barrier-protective and pollutant-binding ingredients may help reduce flare triggers	[24,25,26].
Acne (including chloracne)	Halogenated aromatic hydrocarbons, dioxins, NO_2_	Systemic exposure to dioxins induces chloracne; NO_2_ associated with pigment spot formation	Adsorptive ingredients may help reduce surface pollutant load contributing to inflammation	[27,28]
Skin cancer risk	PM_10_, PAHs, UV radiation	PAHs are photoreactive and carcinogenic, especially with UVA; pollutants activate AhR signaling in skin cells	Protective formulations should limit pollutant–skin contact and oxidative damage	[6,29,30]
Psoriasis flares	CO, NO_2_, NOx, benzene, PM_2.5–10_	Short-term pollution exposure associated with psoriasis exacerbation	Preventive topical strategies may help reduce environmental triggers	[31,32]
Hyperpigmentation (e.g., melasma)	Diesel exhaust particles, PM, ROS-generating pollutants	Pollution increases ROS and melanogenesis; higher incidence reported in polluted regions	Anti-pollution products should combine adsorptive and antioxidant functions	[6,33,34]
Hair loss and impaired growth	PMs, VOCs, NOx, CO, PAHs	Pollutants induce oxidative stress, inflammation, and keratinocyte apoptosis in hair follicles	Scalp products may benefit from pollutant-binding and antioxidant ingredients	[6]

**Table 2:** Natural-derived bioactive molecules regulating skin aging and pollution-induced damage.

Functional Group	Representative Compounds	Natural Source(s)	Principal Mechanisms of Action	Relevance to Pollution-Induced Skin Aging
Flavonoids (polyphenols) [59,60,61]	Quercetin, Epigallocatechin gallate (EGCG)	Green tea (*Camellia sinensis*), various fruits and vegetables	ROS scavenging; inhibition of NF-κB; reduction in lipid peroxidation; modulation of MAPK signaling	Reduce oxidative stress induced by PM and cigarette smoke; prevent collagen degradation
Non-flavonoid polyphenols [56,57,58]	Resveratrol, Ferulic acid	Grapes (*Vitis vinifera*), rice bran, cereals	Activation of Nrf2 pathway; antioxidant enzyme upregulation; anti-inflammatory cytokine modulation	Protect against UV- and pollutant-induced oxidative damage; reduce photoaging markers
Phenolic diterpenes [67,68]	Carnosic acid, Rosmarinic acid	Rosemary (*Rosmarinus officinalis*), sage	Anti-inflammatory activity; inhibition of MMPs; ROS neutralization	Prevent pollution-triggered extracellular matrix degradation
Phytosterols [62,63,64]	Fucosterol	Brown algae	Reduction in ROS production; inhibition of inflammatory mediators	Protect against PM-induced oxidative and inflammatory skin injury
Terpenoids/Essential oil constituents [49]	Patchouli alcohol, Eugenol, Menthol	Patchouli (*Pogostemon cablin*), clove (*Syzygium aromaticum*), peppermint (*Mentha piperita*)	Antioxidant activity; modulation of inflammatory pathways; improvement of barrier function	Improve epidermal barrier integrity and reduce UV/pollution-induced wrinkle formation
Polysaccharides [52]	Dendrobium polysaccharides, Red ginseng polysaccharides	*Dendrobium* spp., *Panax ginseng* by-products	Barrier reinforcement; moisture retention; anti-inflammatory effects; modulation of immune response	Improve xerosis, reduce UV-induced aging, support recovery from pollution exposure
Algal bioactive complexes [59,60,61]	Spirulina–rosemary phenolic complex	*Spirulina* spp., rosemary extract	Reduction of 4HNE and MMP-9; preservation of filaggrin; antioxidant and anti-inflammatory action	Prevent diesel exhaust-induced oxidative and inflammatory damage
Ginsenosides (triterpenoid saponins) [65,66]	Ginsenoside Rb1	*Panax ginseng*	Anti-apoptotic activity; reduction in oxidative stress; mitochondrial protection	Mitigate pollutant-induced keratinocyte damage
Vitamin derivatives [67]	Niacinamide (Vitamin B3), Vitamin C	Various plant and synthetic sources	Enhancement of barrier function; reduction in hyperpigmentation; antioxidant activity	Protect against oxidative stress and pigmentary alterations
AhR modulators (natural small molecules) [42,43]	Tapinarof, Galactomyces ferment filtrate components	Bacterial-derived (Tapinarof), yeast ferment	AhR activation; Nrf2 induction; IL-17 and IL-33 modulation; barrier restoration	Reduce inflammation in AD and psoriasis; counteract pollutant-triggered immune activation
Omega-3 fatty acids [67]	EPA, DHA	Fish oil	Anti-inflammatory effects; inhibition of oxidative stress pathways	Reduce chronic inflammation and oxidative damage related to environmental exposure

**Table 3:** Adsorption Capacities, Origins, and Practical Characteristics of Natural Adsorbents.

Adsorbent Type	Pollutant	Adsorption Capacity (mg·g^−1^)	Removal Efficiency in Topical Formulation	Mechanism	Origin/Source	Extraction/Production	Cost & Availability	Environmental Impact	Ref.
Zeolite (clinoptilolite)	Pb^2+^	120–150	35–45%	Ion exchange	Geological deposits (USA, China, Europe)	Mined, washed, ground, optional thermal/ion exchange activation	Low (<$1/kg); widely abundant	Sustainable mining; low impact if managed	[5,71,79,80]
Zeolite (clinoptilolite)	Cd^2+^	95–130	35–45%	Ion exchange	Geological deposits (USA, China, Europe)	Mined, washed, ground, optional thermal/ion exchange activation	Low (<$1/kg); widely abundant	Sustainable mining; low impact if managed	
Kaolinite clay	PM_2.5_	60–85	40–50%	Surface adsorption	Sedimentary deposits worldwide	Excavated, dried, milled, optional chemical modification	Very low ($0.005–0.46/kg); abundant	Minimal	[73,81,82]
Montmorillonite clay	PM_2.5_	80–110	40–50%	Layered intercalation	Sedimentary deposits worldwide	Excavated, dried, milled, optional chemical modification	Very low ($0.005–0.46/kg); abundant	Minimal	
Activated carbon (plant biomass)	VOCs (benzene, toluene)	90–125	45–55%	Hydrophobic/π–π interactions	Coconut shells, wood, coal	Carbonization + activation (steam/CO_2_)	Moderate; varies by precursor	Energy-intensive; moderate	[75,83,84]
Polyphenol-rich plant extracts	Reactive oxygen species	95–140 (radical scavenging equivalents)	30–45%	Antioxidant radical scavenging	Leaves, seeds, peels	Solvent extraction/enzymatic	Moderate; depends on feedstock	Biodegradable; low impact	[85,86]

## Data Availability

The original data presented in the study are openly available.

## Abbreviations

The following abbreviations are used in this manuscript:

AD	Atopic dermatitis
Ahr	Aryl hydrocarbon receptor
EOs	Essential oils
IL	Interleukin
PAHs	Polycyclic aromatic hydrocarbons
PM	Particulate matter
ROS	Reactive oxygen species
SC	Stratum corneum
UV	Ultraviolet rays
UVR	UV radiation
VOCs	Volatile organic compounds
